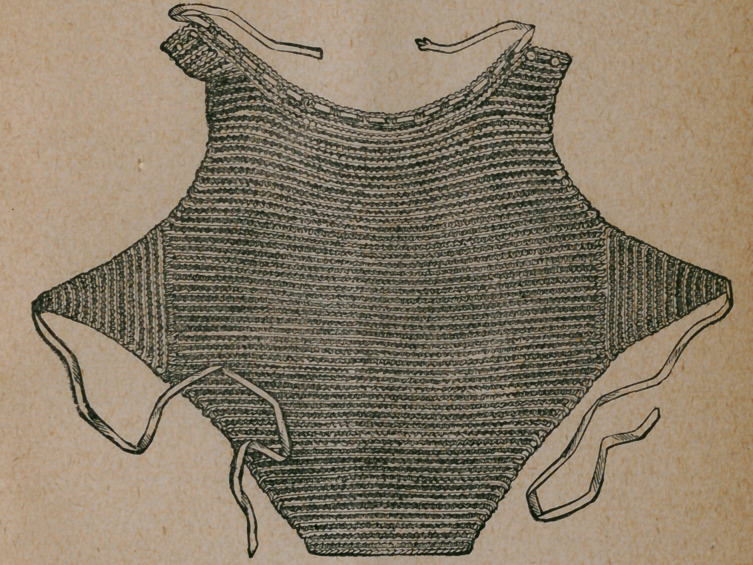# Household

**Published:** 1889-02

**Authors:** 


### HOUSEHOLD.

A KNITTED CHEST PROTECTOR.—Our engraving represents a chest protector which may be easily made by any one who can knit. The material is Shetland wool. The work is plain knitting, the details of which are so plainly shown in the illustration as to require no explanation. For persons with weak lungs, these chest protectors are of great use and comfort in the coldest seasons of the year, as well as in the raw and blustering months of November and March.

CLEANING FURS.—Now that the season has arrived for getting out fur garments, same of our readers will doubtless be glad to hear how such garments are cleaned and renovated in Russia, the country of furs.

Some rye flour is put into a pot and heated upon a stove, with constant stirring as long as the hand can bear the heat. The flour is then spread over the fur and rubbed into it. After this, the fur is brushed with a very clean brush, or, better still is gently beaten until all the flour is removed. The fur thus resumes its natural lustre and appears absolutely as if new.

BIRD'S NEST PUDDING.—Select juicy apples of equal size, pare and remove the cores. Place in a pudding dish ; fill the dish with a nice custard and bake slowly, so as to cook the apples soft.

POACHING EGGS.—Never put an egg, while poaching, over the fire. Let a quart of water come to the boiling point; remove from the fire and then break two eggs into it and in five minutes it will be perfectly poached—a mass of albuminous jelly.

CUSTARD CAKE.—Two eggs, two cups of sugar, two and one-half cups of flour, one-half cup butter, and two heaping teaspoonfuls of baking powder. For the custard: Two eggs, one-half cup of sugar, two cups of milk, two heaping teaspoonfuls of corn starch.

EGG HASH.—Three teaspoonfuls of milk and a pinch of salt to each well beaten egg; to each three eggs add one cupful of chopped, cold, boiled potatoes and one teacupful of chopped fresh meat of any sort, Grease a spider lavishly with meat drippings, and stir constantly until well heated through.

CHICKEN SOUP.—Cut up a chicken into small pieces; put bones and all into a small pot of water. Three carrots cut up, one pint of tomatoes, one teacupful of lima beans, and salt to taste, a little cayenne pepper. One hour before serving, add a pint of rich milk, then thicken with flour, cook for four hours.

HOP YEAST.—Take one cup of fresh hops, three large potatoes, one cup of flour, one soaked yeast cake and one tablespoonful of sugar Boil the hops in a quart of water and drain off ; then scald the flour with the water off the hops; when cool stir in your cake and put away to rise in a warm place; thicken with corn meal and roll out.

ANGEL COCOANUT CAKE.—Two cups of sugar, half a cup of butter, three of flour, one teaspoonful of baking powder, whites of eight eggs, and half a cup of milk. Flavor with vanilla. Bake in jelly cake pans. Spread the top of each with thick icing, then the bottom; let dry and sprinkle thickly with cocoanut. Ice all over and sprinkle with cocoanut.

MUFFINS.—One quart of flour, one pint of warmed milk, one teaspoonful of salt, one-half gill of yeast. Mix at night and beat until light; in the morning drop the dough into buttered cups; let them stand twenty minutes, then bake and serve; to be eaten with butter and sugar, with a little cinnamon, the proportion of a teaspoonful of powdered cinnamon to six of powdered sugar.

GRAHAM BREAD.—Use a little over a quart of warm water, one-half cup of brown sugar or molasses, one-fourth cup of hop yeast, and one and one half teaspoonfuls of salt; thicken the water with unbolted flour to a thin batter; add sugar, salt and yeast, and stir in more flour until quite stiff. In the morning add a small teaspoonful of soda, and flour enough to make a batter as stiff as can be stirred with a spoon; put it into pans in an even hot oven, not too hot at first; keep warm when rising; smooth over each loaf with a knife or spoon dipped in hot water.

CHEAP SNOW PUDDING.—Snow pudding is a delicious dessert and tea dish, but requires a great deal of time to make with gelatine, and it can be made almost as nice and with much less labor by the following recipe: To the whites of three eggs beaten to a stiff froth add two level tablespoonfuls of corn starch, dissolved in a little water, and a pinch of salt, turn this into a half pint of boiling water, using a double kettle; let it cook until stiff, then mold; make a soft custard of three yelks, one teacupful of sugar and one quart of milk; flavor to taste. Serve together when very cold.

## Figures and Tables

**Figure f1:**